# The Prevalence of Depression and Anxiety Among Sickle Cell Disease Patients in King Abdulaziz University Hospital

**DOI:** 10.7759/cureus.18374

**Published:** 2021-09-29

**Authors:** Adel F Al-Marzouki, Norah I Alrefaie, Nora A Aljohani, Raghad A Alandanusi, Abdulrahman A Alghamdi, Osman O Radhwi

**Affiliations:** 1 Hematology, King Abdulaziz University Faculty of Medicine, Jeddah, SAU; 2 Hematology, King Fahd Medical Research Center (KFMRC), Jeddah, SAU

**Keywords:** phq-9, gad-7, health quality of life, anxiety, depression, sickle cell beta-thalassemia, sickle cell disease, saudi arabia

## Abstract

Background

Sickle cell disease (SCD) is a recessive hereditary condition. The physical changes caused by SCD affect the quality of life (QoL) by negatively impacting psychological aspects.

Objective

This study aimed to assess the prevalence of depression and anxiety in SCD patients based on different sociodemographic characteristics in Jeddah, Saudi Arabia.

Method

A cross-sectional study was conducted at King Abdulaziz University Hospital (KAUH) in Jeddah from 13 July to 30 August 2021. The included patients were 18 years of age and above and affected with sickle cell disease. Medical staff interviewed the patients and filled the Patient Health Questionnaire (PHQ)-9 and General Anxiety Disorder (GAD)-7.

Result

One hundred nineteen (119) patients were included in this study. The median age of participants was 32 and mostly male (n=72, 60.5%). The rate of depression was 45.4%. On the other hand, the rate of anxiety was 22.7%. The median of the PHQ-9 score was 8±8 while the median of the GAD-7 score was 5±8. Moreover, the study showed that anxiety and depression in relation to sociodemographics were higher in the patient age groups of 30-34 years old, male, single, unemployed, and with higher education. There was a significant association between depression rate and the two variables: patient employment status (49.3%; p=0.047) and a family history of SCD (51%).

Conclusion

Depression in patients with sickle cell disease is prevalent and correlated to demographic and social factors.

## Introduction

Sickle cell disease (SCD) in Saudi Arabia was first shown in the Eastern province in the 60s [1]. As a response, many regional and national screening studies were done to determine the clinical characteristics and frequency of SCD genes in various regions of Saudi Arabia [2]. Sickle cell disease is a hereditary autosomal recessive condition caused by a mutation in the beta-globin chain genes resulting in what we know as hemoglobin S [3]. Under hypoxic circumstances, usually disc-shaped red blood cells become sickle-shaped, non-deformable, and poorly capable of passing through the small vessels, thus leading to ischemia presented with acute pain and, finally, organ dysfunction [4]. These physical changes have a negative impact on the quality of life (QoL) of SCD patients [5].

Several studies have noted a significant and robust relationship between depression and anxiety, which develops as a result of having a chronic disease such as hepatitis C [6], diabetes mellites [7], and rheumatoid arthritis [8].

Based on two systemic reviews, somatic symptoms, including pain, were strongly associated with increasing the severity of depression in patients with chronic medical illness [9-10]. Moreover, pain is a major and frequent complaint in patients with SCD. In a previous study published in 2018, 89 patients with SCD filled out questionnaires to evaluate anxiety and depression, 36.0% of them were depressed, and 29.2% were anxious. Females were more depressed (56%) and anxious (58%). Single and unemployed patients were more depressed than others. In contrast, the anxiety levels of the patients with high education levels were higher than those with low educational levels [11].

Another study in 2018 from the Eastern province of Saudi Arabia found that depression is prevalent in almost half (48.2%) of patients with SCD [12]. The prevalence was high because the patients in that region suffered from the severest form of the disease, i.e., homozygous SS (HbSS). An additional paper from the Southern Region of Saudi Arabia found that depression prevalence in Jizan and Mahayel Aseer was (56.4%) and (33.3%), respectively [13]; and 10.3% in the rest of the studied cities. A previous study showed that individuals with only a school education level and patients with frequent vaso-occlusive crisis (VOCs) had a more significant risk of developing depression than others. In addition, patients with a low monthly income were at nearly a twofold higher risk of developing depression [12].

A few studies were found that assess the prevalence of depression and anxiety in SCD patients based on different social and demographic characteristics, especially in Saudi Arabia, which is still a gap that needs to be clarified. Therefore, our research aimed to determine the prevalence of depression and anxiety among sickle cell disease patients at King Abdulaziz University Hospital in Jeddah City, Saudi Arabia.

## Materials and methods

Study design and setting

A cross-sectional study was done from 13 July to 30 August 2021 on patients with SCD either homozygous SS (HbSS) or heterozygous S with beta-thalassemia (Hb S/β Thal) who presented to King Abdulaziz University Hospital (KAUH) in Jeddah since 2006. One hundred nineteen patients aged 18 and above were included in the study, with no exclusion criteria.

Data collection instrument

The questionnaire for 119 sickle cells (HbSS) and (Hb S/β Th) disease patients was filled by medical staff (Norah A, Nora A, Raghad A, Abdulrahman A) and was conducted over the phone. Google Forms were used instantaneously, and the data were transformed to Microsoft Excel 2021 (Microsoft Corporation, Redmond, WA). The following variables were extracted from each patient: demographic characteristics (age, gender, marital status, education level, residential status, monthly household income, family history of SCD). Additionally, we administered the Arabic validated questionnaires: Patient Health Questionnaire (PHQ)-9 and General Anxiety Disorder (GAD)-7 (https://www.phqscreeners.com). 

PHQ-9 was used to estimate the depression symptoms in relation to the nine most common symptoms (≤ 4 = low depression, 5-9 = mild depression, 10-14 = moderate depression, > 15 = severe depression) [14]. GAD-7: a seven-item designed to identify anxiety disorder and its relation with functional impairment and disability (≤ 4 = low anxiety, 5-9 = mild anxiety, 10-14 = moderate anxiety, > 14 = severe anxiety) [15].

Analysis

The data was performed using the Statistical Package for the Social Science (SPSS) version 21 (IBM Corp., Armonk, NY). The frequency test was conducted for qualitative data, and the quantitative data were presented as mean ± standard deviation and median ± interquartile range. In bivariate data, the chi-square test was used to assess the differences between categorical variables, and the t-test and Mann-Whitney test were used for non-parametric variables. P-value < 0.05 was considered statistically significant.

Research ethics

The King Abdulaziz University Hospital research ethics committee approved this study (IRB 409-21), and it was carried out according to their guidelines and recommendations. The authors claimed verbal consent from each participant at the beginning of each phone interview.

## Results

Out of 365 patients with SCD registered at KAUH, 119 patients agreed and were eligible to participate. Most of them were male n=72 (60.5%), and n=47 (39.5%) were female. The median age of the whole cohort was 32±9, and n=67 (56.3%) were single. Patients with post-secondary level education were almost half of the cohort (43.7%). Most of the patients in this study had secondary-level education (37%). The monthly household income of most participants (54.6%) was less than 3750 Saudi Riyal (SAR) (1 United States Dollar = 3.75 SAR). The unemployment rate amongst participants was 58%, and 79% had a positive family history of SCD. See Table 1 for additional details.

**Table 1:** Sociodemographics of 119 sickle cell disease (SCD) patients

Variable	No.	(%)
Gender		
Male	72	(60.5)
Female	47	(39.5)
Age		
18-19 years	1	(.8)
20-24 years	28	(23.5)
25-29 year	19	(16)
30-34 years	31	(26.1)
35-39 years	22	(18.5)
Over 40 years	18	(15.1)
Marital status		
Single	67	(56.3)
Married	43	(36.1)
Divorced or widowed	9	(7.6)
Employment status		
Working in the public sector	43	(36.1)
Self-employed	3	(2.5)
Unemployed	69	(58)
Retired	4	(3.4)
Residential status		
Owner	37	(31.1)
Renter	82	(68.9)
Monthly household income		
Less than 1000 USD	65	(54.6)
1000-2000 USD	33	(27.7)
2000-3000 USD	5	(4.2)
More than 3000 USD	16	(13.4)
Education		
Primary level	8	(6.7)
Intermediate level	15	(12.6)
Secondary level	44	(37)
Diploma	10	(8.4)
College level	42	(35.3)
Family history of SCD		
Yes	94	(79)
No	25	(21)

To evaluate the systematic psychiatric aspects of patients with SCD, the PHQ-9 and GAD-7 questionnaires were applied. The median of the PHQ-9 score was 8 (IQR ±8) and the GAD-7 score median was 5 (IQR ±8). Most of the SCD patients had no depression in the PHQ-9 score category, which equals 31%. On the other hand, 47% of the SCD patients have had no anxiety, representing the highest percent according to the GAD-7 score category.

With further evaluation and statistical analysis, scores of ≤10 on both the PHQ-9 and GAD-7 questionnaires were taken as a cutoff value for anxiety and depression [15-16]. Therefore, our study presented that the rate of depression equals 45.4% while the rate of anxiety was 22.7%. Thus, among the sample of depressed SCD patients, 23.5% had only mild depression, 22.7% of the patients had moderate depression, 13.4% had moderately severe depression, while 9.2% of SCD patients were severely depressed. On the other hand, analysis of the GAD-7 questionnaire revealed that 30.3% had only mild anxiety, 13% had a moderate degree of anxiety, and 9.2% had severe anxiety. 

Based on the cutoff percentage of depression rate (45.4%), the rate of depression was most prevalent among the 30-34 age group (27.8%; p=0.902), and 51.9% males (p=0.078) were found to be more depressed than females. Regarding marital status, single patients had higher levels of depression (57.4%, p=0.976) than married and divorced patients. Furthermore, secondary educational levels and college level (40.7%), (30%) (p=0.610) retrospectively were more depressed than subjects who were diploma or with primary and intermediate levels of education. Approximately 63% (p=0.192) of SCD depressed individuals had monthly household incomes less than USD 1000. In terms of residential status, SCD patients in rented houses were found to have a more significant depression of 75.9% (p=0.132) than the owner.

Those who had a positive family history of sickle cell disease were more depressed (88.9%; p=0.016) than those who didn’t have a history of the disease. Moreover, the unemployed patient had a significantly higher depression rate (63%; p=0.047) than the employers. The relation between depression rate and sociodemographic characteristics (age, gender, marital status, employment status, monthly household income, and resident status) was statistically insignificant. In contrast, the depression rate was significantly associated with employment status and the family history of SCD, and the p-values are summed up in Table 2.

**Table 2:** Statistics and p=values of the inferential statistical analysis of depression and anxiety levels with different sociodemographic variables

Variable	Depression		P-value	Anxiety		P-value
	NO.	(%)		NO.	(%)	
Gender			P=0.078			P=0.296
Male	28	(38.9)		14	(19.4)	
Female	26	(55.3)		13	(27.7)	
Age			P=0.902			P=0.613
18-19 years	1	(100)		0	(0)	
20-24 years	13	(46.4)		7	(25)	
25-29 year	8	(42.1)		2	(10.5)	
30-34 years	15	(48.4)		8	(25.8)	
35-39 years	9	(40.9)		4	(18.2)	
Over 40 years	8	(44.4)		6	(33.3)	
Marital status			P=0.976			P=0.7
Single	31	(46.3)		14	(20.9)	
Married	19	(44.2)		10	(23.3)	
Divorced or Widowed	4	(44.4)		3	(33.3)	
Employment status			P=0.047			P=0.348
Working in the public sector	17	(39.5)		7	(16.3)	
Self-employed	3	(100)		1	(33.3)	
Unemployed	34	(49.3)		19	(27.5)	
Retired	0	(0)		0	(0)	
Residential status			P=0.132			P=0.257
Owner	13	(35.1)		6	(16.2)	
Renter	41	(50)		21	(25.6)	
Monthly household income			P=0.192			P=0.327
Less than 1000 USD	34	(52.3)		17	(26.2)	
1000-2000 USD	10	(30.3)		4	(12.1)	
2000-3000 USD	3	(60)		2	(40)	
More than 3000 USD	7	(43.8)		4	(25)	
Education			P=0.610			P=0.103
Primary level	5	(62.5)		4	(50)	
Intermediate level	7	(46.7)		6	(40)	
Secondary level	22	(50)		8	(18.2)	
Diploma	3	(30)		1	(10)	
College level	17	(40.5)		8	(19)	
Family history of SCD			P=0.016			P=0.369
Yes	48	(51.1)		23	(24.5)	
No	6	(24)		4	(16)	

The rate of anxiety is 22.7%, which is for moderately and severely anxious patients. Our study showed that anxiety in relation to sociodemographics was higher in the 30-34 age group (29.6%) than in the other age groups. And, males were more depressed 51.9% than females. Moreover, SCD patients in secondary education level were more anxious (29.6%) than others. However, there was no significant association between the anxiety rate and all sociodemographic characteristics. The p-value is summed up in Table 2.

## Discussion

This study aimed to determine the prevalence and identify the relationship of sociodemographics with depression and anxiety of SCD patients at King Abdulaziz University Hospital, Jeddah, Saudi Arabia. Other researches found that depressed patients will have significantly higher health care utilization and inpatient costs than nondepressed patients [17-19]. So determining the risk factors that increase a psychological disorder in SCD patients will be a chance to decrease the cost and provide much better medical care to these patients.

There is a wide difference in the prevalence that has been reported in the researches across different countries. According to the depression categories and based on the cutoff, the depression rate was 45.4%, which was represented by the (moderate-moderately severe-severe) depression categories; however, the nondepressed is a little higher (54.1%) than depressed patients, which is represented by (nondepressed-mild) depression categories. These findings were compatible with a study conducted in Tabuk that reported a depression rate was 36% and nondepressed SCD patients were 64% [11]. Furthermore, we found that the rate of anxiety in our study was 22.7%, which was less than that reported by Tabuk and Levenson et al. [11,20].

Regarding gender, our results showed the depression and anxiety rate among males was higher than females (p>0.05). These findings are conflict with the results conducted by Tabuk and Gana's study that showed the rate of depression is higher in females [11,21]. Similarly, the results reported by the Southern Region of Saudi Arabia also show the rate of depression and anxiety is higher in females [19]. However, these results failed to indicate any statistical significance in our study.

Our study showed the median age of the whole patient was 32±9. Hence, the prevalence of depression rate was highest among the 30-34 age group. In contrast, other researchers (Adzika et al.) reported a significantly high depression rate in the 40 to 49-year-old age group [21].

According to marital status, the majority of the SCD patients were single. Therefore, the single patients had higher levels of depression (57.4%) and anxiety (51.9%) than married and divorced patients. In addition, 31 single patients were depressed, which represented (46%) of the total 67 single patients who participated in our research. This is in line with another study done in New York, which found that social support was associated with high self-esteem, which in turn increased optimism and was related to decreased depression [22].

Patients with sickle cell disease who had higher education levels such as secondary and college educational levels were more depressed and anxious. Similar results were revealed by a study in the Tabuk region of Saudi Arabia [11]. These findings contradict Ghana’s study that reported a significantly high depression score for individuals with only primary education [21]. According to the employment status in our results, 70% of unemployed people have more anxiety. In addition, 63% of unemployed individuals had higher levels of depression than others, which revealed a statistically significant association(p<0.05). A study published in Jamaica about the prevalence of depression in SCD patients reported that unemployment was linked to depression in people with SCD, which is similar to our study [23].

More than half the patients (48) who had a positive family history (94) are considered depressed (p-value<0.5), and they have faced difficult psychological moments in their life. Moreover, a Saudi paper published in 2018 showed the same results that we extracted, as it reported that sickle cell patients face psychological obstacles throughout their life due to the disease [11]. However, the relation was not entirely significant. Unfortunately, it was concluded that having a first-degree relative in the family with an anxiety-related illness increases the likelihood that a new family member will develop anxiety problems, based on a case-control study published in 2008 [24].

The limitation of our research was a phone-based questionnaire. Hence, we didn’t see the patient's reactions and determine whether the answers are truthful. Furthermore, the coronavirus disease 2019 (COVID-19) pandemic may affect the percentage and increase the incidence of anxiety [25]. In addition, we recognize that some patients may intentionally avoid answering some suicidal and harm-self questions, which may be related to religious aspects or due to fear of revealing their thoughts. Therefore, we recommend clarifying the pathophysiology of depression diseases, and we think that a clinical interview compared to the phone-based questionnaire may have been a more accurate approach.

## Conclusions

This study aimed to obtain the prevalence of depression and anxiety in sickle cell patients at King Abdulaziz University Hospital in Jeddah. The results showed that nearly half (45.4%) of the participants in this research study have depression. However, male patients had slightly higher rates of depression and anxiety than female patients. Moreover, the research showed a significant relationship between depression rates and employment status. More research like this is needed in Saudi Arabia's western region to obtain better insight into the psychosocial impact of SCD patients, gather more data, and formulate an appropriate plan to deal with psychological and social issues.
